# Is corporate green investment a determinant of corporate carbon emission intensity? A managerial perspective

**DOI:** 10.1016/j.heliyon.2023.e22401

**Published:** 2023-11-17

**Authors:** Sisi Zheng, Shanyue Jin

**Affiliations:** College of Business, Gachon University, Seongnam, 13120, Republic of Korea

**Keywords:** Green investment, Carbon emission intensity, Management shareholding, Top management team stability, Environmental background executive power

## Abstract

In recent years, the swift growth of industrialization and globalization has exacerbated serious problems, such as climate change resulting from carbon emissions. As the largest carbon emitter globally, China is striving to explore a low-carbon economic transition path to alleviate environmental issues and ensure long-term development. The relevance of investing in green projects is highlighted in the UN's Sustainable Development Goals. Exploring the effects of green investments on carbon emissions by Chinese companies and the dynamics between the two is crucial for China’s green transition. This study investigates whether companies can reduce their carbon emissions per unit of activity by investing in green projects. Additionally, it evaluates how management decisions influence the effectiveness of these investments in reducing emissions. The study samples firms from the Stock exchanges in Shanghai and Shenzhen for the period 2010–2020. Data are obtained from the China Stock Market and Accounting Research (CSMAR) and DIB, etc. Findings indicate that firms can reduce their corporate carbon emissions by increasing their green investments. Furthermore, factors like management shareholding, etc help strengthen the link between corporate green investment and carbon intensity. This study not only enhance the theoretical comprehension of the link between green investment and carbon emission reduction but also enriches stakeholder theory. The instrumental role of management in urging firms to decrease their carbon emissions via green investments is evident in practice, offering a roadmap for businesses to prioritize green investment choice and create a pathway to a carbon-neutral economy.

## 1 Introduction

Rapid industrialization and modernization consume large amounts of fossil energy, contributing to problems such as severe greenhouse gas emissions [1] and PM_2.5_ pollution [2] on a global scale. Global warming has also caused a series of extreme events, such as glacial melting, forest fires, strong extreme mid-latitude storms, lake heat waves, and extreme precipitation in urban clusters [[3], [4], [5], [6]]. Extreme weather events have put the need to reduce carbon dioxide (CO_2_) emissions to zero and adapt to climate change in the spotlight [7]. In total, 131 countries have set 2050 as the target date for achieving carbon neutrality [8]. The international community has agreed on the common aim of achieving net-zero CO_2_.

China has changed into the largest energy consumer and emitter in the world as its economy has risen in recent years [9,10]. The environmental degradation caused by carbon emissions may endanger the sustainability of China's social and economic development [11]. To meet international environmental responsibilities, China pledged to reach peak carbon levels by 2030 and attain carbon neutrality by 2060 [12]. Many domestic and foreign scholars regard carbon emissions as an important research topic, and some scholars have proven that carbon emissions affect the realization of sustainable development goals [13]. To attain a low-carbon economy, it is crucial to learn what drives carbon emissions. Numerous academics consider carbon emission efficacy as an essential indicator for measuring carbon emissions. For example, Xu et al. found that green technological innovation positively impacts carbon emission efficiency [14]. Ray et al. demonstrated that lockdown measures brought about by the COVID-19 pandemic lowered global carbon emissions but were not sustainable [15]. For sustainable development, the relevant authorities must implement measures in terms of transportation, climate, and environmental policies that contribute to global carbon emissions [15]. Recently, the low-carbon city pilot policy [16] and carbon emission trading policy [17] implemented by the Chinese government has helped improve carbon emission efficiency. Simultaneously, major measures have also been taken in the transformation of renewable energy [18], such as increasing government subsidies and R&D investments [19]. Although China has taken many measures to transform renewable energy, challenges with backward technology and insufficient investment in renewable energy remain [20]. This has hindered the pace of the country’s green transition. Therefore, to ensure environmental sustainability across China, green investment is pivotal to reducing carbon emissions [20]. The function of ecological investments, especially to reduce environmental pollution, is highlighted several times in the United Nations Sustainable Development Goals agenda [21]. For example, Sustainable Development Goal 12 aims to promote investment in climate finance to maintain climate stability and a worldwide shift to a carbon-neutral economy. In this regard, Green Climate Fund can help lowering carbon output and adapting to climate change [22]. In addition, green investment can advocate for the expansion of renewable energy sources and related infrastructure, contributing to the achievement of SDG 7. Green investments, as proven by Lyeonov et al. (2019), can raise renewable energy's proportion of total energy use while decreasing emissions of greenhouse gases [23]. But there is no agreement on how green investments affect emissions of greenhouse gases. The negative correlation between green investment and carbon emissions, as indicated by Shen et al. (2021), can be mitigated with the use of this factor [24]. In Ren's opinion, the effect of green investment on contamination of the environment varies non-linearly with system quality [25]. Therefore, it is critical to find out how eco-friendly spending affects efforts to cut down on greenhouse gas using Chinese firms as research samples.

The thesis of the "upper layer" emphasizes the importance of top-level managers in business operations management because of their professional knowledge and experience [26]. Therefore, it is important for executives to study corporate investment decision-making behavior. Using Chinese enterprises as an example, scholars studied the impact of the executive equity incentive plan (EEIP) on green innovation and obtained positive conclusions [27]. From the perspective of executive gender, some scholars have also demonstrated that female CEO representation has a strong detrimental influence on corporate green innovation [28]. Additionally, some scholars have studied top management teams, mostly starting with behavioral decision-making and mobility. For example, Cho et al. believed that top management team mobility reduce R&D intensity and has a detrimental influence on an enterprise's long-term investment [29]. CEO authority was evaluated as a moderating factor, and Javeed et al. discovered that it moderates the beneficial association between CSR and top-level management-driven innovation [30]. Nassani et al. proposed that stakeholders focus more on resource preservation through environmental management [31]. Although previous studies recognized the significance of CEOs in green corporate decisions, few explored this angle as a moderating factor to understand the dynamics. That gap in the existing literature is what this investigation sets out to address.

This study introduce several innovations. First, considering that enterprises are the main participants in economic activities and carbon emissions [27], previous studies have mostly used provincial data samples [28]. In contrast, this study gathers corporate-level carbon emissions data from various source, including corporate sustainable development reports and annual reports, presenting a more innovative and practical approach to data collection. Second, in the context of variable selection, previous studies mostly focused on total carbon emissions [32], carbon emission efficiency [14], and carbon emission policies [16]. Here, we focus on corporate carbon intensity, which represents the amount of carbon dioxide released per revenue earned. This metric is selected for its relevance as a significant indicator of environmental quality and progress toward carbon neutrality goals. Additionally, our study uniquely adopts a managerial perspective to delve deeper into the mechanics of how corporate green investments influence carbon emission intensity. In identifying relevant indicators for gauging the consistency of top management teams, rather than resorting to the commonly-used dummy variables from prior research to indicate executive turnover, we have applied the concentration algorithm of the Herfindahl index to the stability factors of all executive team members over the last three years.

Given the state of the current research, this study considers Chinese listed companies as research subjects to empirically explore whether corporate green investment reduce corporate carbon emission intensity and the specific mechanisms behind this. This study uses signal transmission, environmental economics, resource-based, and higher-order theories. Our findings offer micro-level evidence, addressing a research gap in the field. Simultaneously, we investigate the influence of variables, such as management shareholding, on this effect from a management perspective. This examination broadens the literature concerning corporate green investment and carbon emissions reduction. It further enriches the knowledge of stakeholders, environmental governance, green development, and other related theories. Moreover, it provides effective theoretical guidance for enterprises to conduct green investments and reduce carbon emissions.

The main purpose of this study is to explore the factors affecting the reduction of carbon emission intensity of Chinese enterprises, and to find feasible paths for Chinese enterprises to save energy and reduce emissions. This study takes green investment as the entry point, in order to analyze whether the level of green investment of enterprises is an important factor in reducing the carbon emission intensity of enterprises from the micro level. At the same time, considering that management plays an important role in corporate business decision-making, this study intends to explore the role mechanism of corporate green investment in reducing corporate carbon emission intensity from three perspectives of management. Finally, we get whether management shareholding, executive team stability and environmental background executive power can increase or slow down the relationship between corporate green investment and carbon emission intensity. Because the main body of the authors' study is enterprises, they consider that there may be heterogeneity among enterprises in different years and different industries and individual characteristics among enterprises, as well as the possibility of heteroskedasticity. Based on this, a high-dimensional fixed-effects model was selected for analysis in this study, while considering the robust standard error of heteroskedasticity.

Since the promulgation of the Kyoto Protocol, academics have been concerned about carbon emissions for a long time. Many scholars have also analyzed how to achieve China's carbon dioxide emission reduction target from the perspective of investment, especially green investment. However, scholars have given different answers to the relationship between green investment and carbon emissions: one viewpoint is that the two have a linear relationship, another viewpoint is that the two have a non-linear relationship, and other scholars believe that green finance has a heterogeneous impact on carbon emissions. However, there is still room for expansion in the research results of scholars. First, from the research perspective, most scholars focus on the green financial perspective when studying the impact of green investment on carbon emissions, and there is little literature that directly studies the relationship between green investment and carbon emissions. Second, from the perspective of research content, the current research on the relationship between green investment and carbon emissions has not been uniformly finalized, and further discussion is needed. Thirdly, from the point of view of using data, at present, due to the fact that Chinese enterprise-level carbon emission data is not easy to obtain, most scholars use provincial panel data, and there is still a large number of gaps in terms of the results of the argumentation through enterprise-level data. The findings of the study can also further enrich and quantify the carbon accounting of enterprises. In addition, this study creatively starts from the management perspective by incorporating management shareholding, executive team stability and environmental background executive power with both into a unified analytical framework, which also bridges the research gap on the role of managers in corporate investment decisions and energy saving and emission reduction.

The remainder of the paper is structured as follows: Section 2 presents the theoretical background and analyzes and formulates the hypotheses with respect to the research questions. Section 3 covers the methodology. Section 4 presents the empirical analysis. Section 5 discusses the results of the study, the conclusions, and the limitations and future perspectives.

## 2 Literature review and hypotheses

### 2.1 Green investment and carbon emission intensity of enterprises

According to environmental economics theory, balancing and coordinating the economic-environmental relationship is pivotal, as these two domains influence each other [33]. Elkington identified the economy, society, and environment as the "triple bottom line," essential for the sustainable development of enterprises [34]. Carbon emissions, especially in the manufacturing sector, pose a significant threat to environmental quality and productivity [20]. Historically, scholars have examined various economic factors influencing carbon emissions, including green investment [35], natural resource [36], urbanization [37], foreign direct investment [38], and green finance [39]. Currently, there is not a unified definition of green investment. Eyraud et al. defined it as capital investment in renewable and energy-saving technologies [40]. Scholars like Li et al. referenced Krushelnytska's introduction to green finance [40]. It is argued that green investment extends beyond technology investments focused on improving energy use efficiency and embracing renewable energy. It encompasses ventures that foster waste management [20]. Furthermore, green investment is seen as vital to carbon emission reduction [20]. Guo et al. discovered that spending on renewable energy and the energy sector leads to a dramatic decrease in greenhouse gas emissions [41].

The EKC theory, or Environmental Kuznets Curve, states that, at the end of industrialization and the entire post-industrialization stage, the technological effect of technological progress may reduce unclean industrial output. This implies that emissions can significantly minimized [ 42]. In the EKC analysis, almost all related studies on energy consumption control asserted that renewable energy source taking over for traditional ones is beneficial for reducing greenhouse gas emissions [42]. This further underscores the importance of green investment in renewable energy. Sachs et al. examined how green projects and investments can help achieve the SDGs [21]. Azhgaliyeva et al. based their research on private investment, claiming that it can reduce global carbon emissions while fostering global growth in a green and low-carbon manner [43]. Based on prior research and the above analysis, this study presents Hypothesis H1 to further validate the micro-level association between company green investment and corporate carbon emissions reduction.

Hypothesis 1 (H1). Corporate green investment negatively impacts corporate carbon intensity*.*

### 2.2 Moderating effect of management shareholding

The stewardship theory holds that self-disciplined management and the stakeholders' goals are consistent, and will maintain the position and identity of "good stewards.” When management with limited rationality does not discipline itself, it deviates from the position of "good stewardship,” which is reflected in the agency conflict between management and company stakeholders. Major shareholders are prone to using their controlling positions to extract personal benefits, which can lead to agency problems [44,45]. Companies with controlling shareholders tend to engage in fewer CSR activities, thereby supporting the curing effect of controlling shareholders [44]. In contrast, Long-term incentives like executive stock ownership (ESO) allow executives and companies to split the profits and losses [46]. Incentivizing management with equity can promote the convergence of interests between the management and shareholders. It alleviates agency conflicts and information asymmetry problems, while allowing management to choose to increase their green innovation investment because of their proactive care about the company's future viability [27].

Based on signaling theory, management ownership can transmit high-quality information to external stakeholders of the enterprise [47]. CSR has become an important criterion for measuring the abilities of executives and employment of enterprises [48]. Therefore, as the results of Chen et al. showed, executives prefer to spend money on CSR to avoid being fired and have a broader scope for promotion [49]. A favorable incentive environment can also mitigate the external drivers of executive shortsightedness, stimulate management to improve their capabilities, and reduce potential shortsighted behavior. Management shareholding, through the incentive of balanced equity, ensures executives’ independence in actively implementing CSR decisions, establishing a system of green governance that works, and ensuring sustainable growth over the long term [50]. This leads us to the hypothesis that we present below: H2, Hypothesis 2 (H2). *Management shareholding can further enhance the negative impact of corporate green investment on corporate carbon intensity.*

### 2.3 Moderating effect of the stability of top management team

The importance of the top management team in modern corporate innovation should be emphasized [51,52]. The upper echelons theory underpins top management team studies [26,53]. Besides, the theory also showed that the variety of traits of the top management team is an important determinant of the long-term success of a business and investment decision-making [54]. This heterogeneity includes the individual backgrounds of team members [55], which can reflect their investment decision-making behavior. The top management team's strategic decision-making is crucial to the company's success. To fully exploit the favorable role of the senior management team, its tenure should be extended [54].

The resource-based theory views team cohesion as a pivotal strategic resource for an enterprise. A cohesive and stable executive team often paves the way for effective teamwork and unity among its members. Such stability in a team aids executives in rapidly reaching consensus on intricate green investment decisions, like amplifying investments in innovation [56]. For instance, Agarwal et al. contended that the stability of the executive team stands as a critical team attribute. They noted that executive departures can fracture team cohesion, trigger cognitive disagreements among members, and disrupt the consistency of corporate strategies and business decisions [57]. Concurrently, such disruptions can breed managerial myopia, leading to an oversight of environmental decisions, which can ultimately jeopardize a company's long-term growth. Drawing from the above theoretical discussion and historical research, we propose Hypothesis 3 (H3):

Hypothesis 3 (H3). There may be a greater negative impact of corporate green investment on business carbon intensity if the top management team remains stable.

### 2.4 Moderating effect of executive power with an environmental protection background

The upper echelons theory posits that, given the same environment, the experience and values of corporate executives play a pivotal role in dictating how organizations respond to regulatory pressures in varied ways [26]. Executives with experience in environmental protection typically exhibit heightened environmental awareness. Similarly, the attention-based view suggests that the outcomes of organizational decision-making are influenced not just by the personal attributes of decision-makers, but also by cognitive aspects, such as where they focus their attention [58,59]. Consequently, executives who have a background in environmental protection tend to be more prone to prioritize the corporate environment, facilitating greater investment in environmental expenditures [60].

According to the resource-based view theory, firms should increase environmental investment and quicken the velocity of the green transition [61]. Velte et al. (2020) argued that executives from environmental backgrounds can reduce the risk associated with corporate environmental investments owing to their specialized environmental knowledge and experience. This, in turn, encourages companies to augment their environmental investment expenditures, thereby enhancing their commitment to corporate environmental responsibility [62]. Finkelstein highlighted that power is an asymmetric relationship between individuals [63]. Those holding greater power often have a more dominant voice compared to their counterparts with lesser power, and they might even assert their perspectives as the ultimate decision, especially in the face of dissent. Atif et al. suggested that the influence of executive power affects a firm's commitment to sustainable investment [64]. Consequently, as executives with environmental backgrounds ascend within their teams and amass power, their capacity to sway other team members also grows. This allows them to effectively convey their environmental concerns to the entire executive team, prioritizing it as a central issue. Given the preceding theoretical analysis and literature insights, we proposes H4:

Hypothesis 4 (H4). Environmental background executive power can further enhance the negative impact of corporate green investment on corporate carbon intensity.

**Fig. 1:**
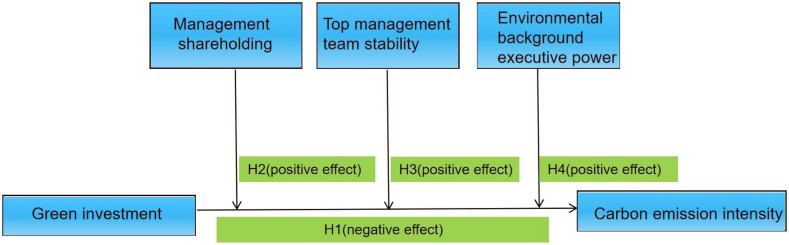
The study model.

Fig. 1 is the research model of the study.

## 3 Research method and design

### 3.1 Data and samples

In 2010, the former Ministry of Environmental Protection developed the "Guidelines for Environmental Information Disclosure of Listed Companies" to enhance increase corporate environmental information transparency. This study posits that, following the release of these "Guidelines," Chinese enterprises have become more engaged in environmental protection and have started disclosing more carbon information. Given the limited green investment data available for enterprises prior to 2010, we designated the sample period from 2010 to 2020.

Moreover, the Stock exchanges in Shanghai and Shenzhen are significant in size, reflecting the dynamics of the Chinese economy and policy direction. Since 2006, these stock exchanges have introduced social responsibility regulations, requiring listed companies to disclose environmental protection information. As a result, focusing on companies listed on these exchanges streamlines the data collection process, ensuring that our study relies on accurate and comprehensive data.

Specifically, we consult the CSMAR database for information between the years of 2010 and 2020. We exclude companies undergoing special treatment, special treatments, and particular transfers from our sample. Concurrently, we remove data from the financial sector and any data sets with missing values. To minimize the impact of outliers, we winsorize the variables at the 1 % levels on both ends, excluding dummy variables. We also log-transform the continuous variables to mitigate heteroskedasticity. After these data adjustments, we obtain a total of 8034 valid samples. We employ Stata 17.0 for data cleaning, analysis, and to deliver statistical results.

The green investment data for Chinese enterprises is sourced from cninfo.com, financial report notes, and social responsibility reports. Reports on corporate social responsibility, sustainability, and the environment are the primary source for information on carbon emissions. The data on green investments and carbon emissions used in this study was crawled manually [65,66]. Data on management shareholdings is procured from the Dibo database [67]. Information on top management team stability and executive environmental background power is gathered from annual corporate reports [68,69]. Lastly, the CSMAR database is instrumental in obtaining data on company financial metrics and related variables.

### 3.2 Variables and their measurements

#### 3.2.1 Dependent variable

Carbon emission intensity of the enterprise, which is defined as emissions of greenhouse gases produced by the company per unit of income [70] and is a reliable indicator reflecting climate risk. To improve the readability of the data, the carbon intensity is determined according to Chapple and colleagues' methodological formula [71] and multiplied by 100000.

Equation (1) refers to the studies of Kim et al. [70] and Zheng et al. [72], who both used corporate carbon emissions divided by corporate sales revenue or total assets as the basis for measuring the carbon intensity at the corporate level. In this study, in order to reflect the carbon emissions per unit of income of enterprises, the carbon emissions of enterprises divided by the business revenue of enterprises were chosen as the calculation standard.

(1) $$\text{Enterprise}\;\text{carbon}\;\text{intensity}=\frac{\text{Enterprise}\;\text{carbon}\;\text{emissions}}{\text{Enterprise}\;\text{main}\;\text{business}\;\text{income}}\times 100000$$

This study calculates corporate greenhouse gas emissions in accordance with the standards established by the internationally accepted "Greenhouse Gas Protocol " [70,73]. The GHG Protocol categorizes corporate carbon emissions into direct emissions from resource owned or controlled by the business corporation; emissions from the use of commercial electricity and heat; and all additional indirect emissions from source beyond the corporation's direct control. We chose the mandatory disclosure part of the GHG Protocol as the sample scope of the study, removing all additional indirect emissions beyond the company's direct control.

At the time of data collection, we used publicly available resource, such as annual reports and sustainability reports, to collect information that was released by corporations themselves. We also get carbon emissions data by calculating according to the IPCC Guidelines for companies that simply reveal their corporate consumption of fossil fuels, electricity, and heat.

For enterprises that did not directly disclose carbon emissions data but only disclosed fossil energy consumption, electricity consumption, and heat consumption, this study referred to the study by Guan et al. [74] and used equation (2). For enterprises that consumed fossil energy, carbon emissions data were obtained by multiplying the activity level data for the fossil fuel consumed by the emission factor for that fossil fuel; for enterprises that incurred electricity consumption, the purchased electricity was used to multiply by the average grid emission factor for the region in which the enterprise was located; and for heat consumption, it was necessary to use the emission factor determined by China's uniform regulations. The specifics are outlined below.

(2) $$E_{i}=AD_{i}\times EF_{i}$$

AD is the activity level data for consuming this fossil fuel and is obtained by multiplying the fuel consumption with its average low calorific value, which reflects the situation related to the fossil fuel consumption of the businesses, and EF represents the emission rate of fossil fuels. Data from the "Guidelines" released by the National Development and Reform Commission were used to determine value of low heat production on average and emission factor. Information on fuel usage is compiled from publicly available sources.

When calculating electricity consumption, AD represents the electricity consumption of a company and the National Climate Change Strategy Research and International Cooperation Center supplies this data.

For heat consumption, the emission factors, derived in line with national uniform regulations, should be used. Specifically, the factor is $0.11\text{tC}O_{2}/\text{GJ}$.

#### 3.2.2 Independent variable

Taking from Eyraud et al.'s definition of green investment [40], this study encompasses all expenses linked to environmental protection as green investments. Building on the studies by Dang and Zheng, this study considers the cumulative amount of green investments made and expended by companies as the overall environmental protection investment [71,75]. To offset scale effects and improve data clarity, multiplying 100 by the result of dividing the entire environmental expenditure by the total asset value, resulting in a measure for green investment [71].

In calculating corporate green investment, this study refers to the definition of green investment by Zhang et al. [74] and uses the sum of environment-related capitalized and expensed expenditures as the object of the study. Capitalized environmental investment refers to the new investment in the current year, which is counted according to "under construction" in the annual report, including pollution prevention, clean production, wastewater treatment, desulfurization and denitrification, and energy saving. Expensed environmental protection investment mainly includes green fees, sewage charges and environmental taxes from 2018 onwards, which are summarized from "administrative expenses" and "business taxes and surcharges" in the annual report. Equation (3) refers to the calculation of green investment by Dang [71], which is obtained by dividing the environment-related capital expenditure by the total assets at the end of the year and multiplying it by 100, which also provides support for replacing the range of independent variables in the subsequent robustness test. Specifically,

(3) $$\text{Green}\;\text{investment}=\frac{\text{Total}\;\text{environmental}\;\text{investment}}{\text{Total}\;\text{assets}\;\text{at}\;\text{year}-\text{end}}\times 100$$

#### 3.2.3 Modulated variables

Management shareholding serves as the initial moderating variable in our research. Within the context of this study, the term "management" aligns with Yan et al.'s definition, encompassing "directors, supervisors, and senior managers" [76]. Management shareholdings can be thought of as a percentage of the total number of shares outstanding at the conclusion of the fiscal year that is held by management.

Equation (4) refers to Sarhan and Al-Najjar's study [77] and Javeed & Lefen's study [78], where management shareholding is calculated using the percentage of the total number of shares owned by management. Specifically,

(4) $$\text{Managemen}t^{\prime }s\;\text{share}\;\text{holding}=\frac{\text{Managemen}t^{\prime }s\;\text{share}\;\text{holding}\;\text{at}\;\text{the}\;\text{end}\;\text{of}\;\text{the}\;\text{year}}{\text{Total}\;\text{number}\;\text{of}\;\text{shares}}$$

The stability of the top management team (TMT) serves as the second moderating variable. Drawing from Zhu et al., this study measures the stability of a company's top management team, formulating a stability index (Stability) that hinges on both the position and duration of team members to evaluate the prolonged stability of the TMT [79]. In line with Zheng et al., this study defines the TMT to encompass all directors and top-level managers. However, it excludes independent directors and supervisors mentioned in the annual report, such as chairpersons, directors, general managers, and vice-presidents [80]. Given that the typical tenure for corporate executives spans around three years, this study compiles a roster of corporate executive team members from 2010 to 2020. It then gauges the shifts within the corporate executive team over a dynamic three-year window. To ascertain the stability of corporate executive teams, this study employs the subsequent formula:

(5) $$X_{j,i}=\text{Positio}n_{j,i}\times T_{i}\times \text{Ad}j_{i}=\frac{m_{j,i}}{\sum_{j=1}^{n}m_{j,i}}\times \frac{t_{i}}{\sum_{i=1}^{k}t_{i}}\times \sqrt{\frac{1}{\sum_{j=1}^{n}{\text{Position}}_{j,i}^{2}}}$$

(6) $$\text{Stability}=\sum X_{J}^{2}=\sum {\left(\sum X_{j,i}\right)}^{2}$$

In this equation, $X_{j,i}$ represents the stability factor of corporate executive j in year i, and n denotes the number of corporate executive teams in year i. $\text{Positio}n_{j,i}$ represents the position weight of corporate executive j in year i. $T_{i}$ is the time weight in year i. $\text{Ad}j_{i}$ denotes the adjustment term of the number of positions in year i to eliminate the systematic influence caused by the difference in executive team size in different years. When calculating the position weights $\text{Positio}n_{j,i}$, we consider that corporate executives with different positions have different powers and influence. The board of directors makes long-term decisions for the corporation, while the executive team implements those decisions. Therefore, this study assigns different values to corporate executives in different positions. The (vice) chairperson is assigned a value of two ($m_{j,i}$ = 2), and other executives are assigned a value of one ($m_{j,i}$ = 1). In particular, this study considers the impact of top positions on stability when executives serve concurrently in the company. Simultaneously, the impact of a change in top executives on the business intensifies over time. Therefore, when calculating the time weight $T_{i}$, this study takes three years as the period and assigns $T_{i}$ in the order of increasing by one unit from farther to nearer years ($t_{i}$ = 1, 2, 3), thus taking the values of 1/6, 1/3, and 1/2 for the past three years, respectively.

In the specific calculation of the stability of the company's executive team, this study first uses Equation (5) to calculate the stability factor $X_{j,i}$ for each year of the company's executive j. Second, using Equation (6), the stability factor X_j_ for the past three years of executive j is obtained by summing, and then the stability factor X_j_ for all members of the company's executive team in the past three years is summed by squaring according to the concentration algorithm of the Herfindahl index to obtain the stability variable (Stability) of the executive team. Therefore, a higher stability value indicates a higher level of stability among a company's executive teams.

The third moderating variable is environmental background executive power. Executives are those whose names appear on the company's annual report, according to He et al. [81]. Executives' resumes are screened to identify those with prior environmental work experience. This includes experience such as holding positions in governmental environmental protection departments or environmental associations, participating in environmentally-related projects, earning academic certificates, or possessing patented technologies pertinent to environmental protection. Learning from Ke et al. [82]: First, based on the ranking of executives with environmental protection backgrounds in the entire executive team, we calculate the power score of an executive according to Equation (7). In Equation (7), Tpower represents the power score of a certain executive, Trank denotes the ranking of the executive in the list of executive teams disclosed in the annual report, and Tsize serves as a proxy for the size of the executive team of the company in that year. Second, considering that some companies boast multiple executives with environmental backgrounds, this study aggregates their power scores and divides the total by the sum of all executive scores. After adjusting for potential discrepancies owing to varying team sizes and the presence of several executives with environmental expertise, a consolidated power score, termed EPower, is determined for executives with environmental backgrounds.

(7) $$\text{Tpower}=1-\frac{\left(\text{Trank}-1\right)}{\left(\text{Tsize}-1\right)}$$

#### 3.2.4 Controlled variables

Control variables in this analysis include age of the company (Age), top ten ownership (Top10), ownership nature (Soe), and cash flow ratio (Cashflow). Other metrics include leverage ratio (Lev), return on assets (Roa), and total asset growth rate (Tagr) [83,84]. Considering that businesses in various industries or different years might experience variations in their sector's carbon intensity owing to shifts in industry policies and the broader macro-environment, we incorporated industry (Industry) and year (Year) effects, setting them as dummy variables. A business is allocated a value of 1 if it belongs to the specified industry or year; otherwise, it is assigned a value of 0. Table 1 provides detailed explanations and formulas.

**Table 1:** Variable definitions.

Variables	Symbols	Names	Measurements
Dependent variable	Cei	Carbon intensity	Enterprise carbon emissions/Enterprise main business income
Independent variable	Cgi	Green investment	Total investment in environmental protection/Total assets at the end of the year
Moderating variables	Mshare	Management ownership	Shares owned by management at year's end as a percentage of total shares owned at year's end
	Stability	Stability of the top management team	Top management team stability considering position and year weighting over a three-year cycle
	Epower	Environmental protection background Executive power	Power score of executives with environmental Background/Sum of executive scores
Controlled variables	Size	Enterprise size	Annual total assets logarithmically expressed
	Lev	Assets and liabilities	Total liabilities/Total assets
	Roa	Return on assets	Net profit divided by the average balance of total assets
	Tagr	Growth rate of total assets	The total assets of the current year to the total assets of the previous year minus 1
	Cflow	Cash flow ratio	Operating cash flow as a percentage of total assets
	Tat	Total asset turnover	The beginning and ending total asset average as a percentage of sales for the period
	Top10	Concentration of ownership	Shareholding ratio of the top ten shareholders
	Soe	Nature of property rights	= 1 if state-owned; otherwise = 0
	Age	Business age	ln (observation year - registration year +1)
	Industry	Industry	Dummy variable for industry; if the value is 1, the industry is present in the corresponding year; else, the value is 0.
	Year	Years	Time dummy variable, where 1 indicates the current year and 0 indicates all other years.

This thesis explores the impact of corporate green investment on carbon emission intensity at the micro level. Referring to previous studies on the determinants of carbon emissions, such as Chen et al. [85] and Chen et al. [86], which concluded that equity concentration has a negative impact on corporate fulfillment of environmental responsibility, and that the nature of equity also exerts a heterogeneous effect on corporate environmental behavior, this study includes the proportion of the top ten shareholders' shareholdings and the nature of equity as control variables. In addition, because it is firm-level data, we again control for firm-specific relevant variables, drawing on the studies of Albitaret al. [87] and Gerged et al. [88] on carbon emissions. These include, firm size as measured by the natural logarithm of total assets (Size); leverage as measured by total liabilities divided by total assets (Lev); firm profitability as measured by net profit divided by average total assets (Roa) and an indicator of the growth rate of total assets that reflects the growth capacity of the firm (Tagr). This study also refers to Lv & Bai's [89] study on carbon emissions trading in China to include operating cash flow as a percentage of total assets as a measure of cash flow ratio (Cflow) and firm age (Age) as control variables. Industry and year were also controlled.

### 3.3 Construction of a model

#### 3.3.1 Baseline model

Based on the work of Ren et al., we develop the following basic model to examine the correlation between green investment and the carbon intensity of enterprises [25]. Equation (8) referred to Chen & Ma's study on the relationship between green investment and environmental performance [56]. Meanwhile, a linear model was used with reference to Ren et al.'s basic model on green investment and environmental pollution [25].

(8) $$\text{Ce}i_{i,t}=\alpha_{0}+\alpha_{1}\text{Cg}i_{i,t}+\Sigma \text{Contro}l_{i,t}+\mu_{i}+\gamma_{t}+\varepsilon_{i,t}$$

The dependent variable is the company's carbon emissions intensity (Cei). The independent variable is corporate green investment (Cgi). Control factors affect businesses’ carbon emission intensity. Furthermore, to offset the impacts of individual company characteristics and year features, we choose a two-way fixed-effects model where $\mu_{i}$ represents individual effects and $\gamma_{t}$ represents year effects. $\varepsilon_{i,t}$ represents the randomized perturbation term. In view of the possible heteroscedasticity problem, we also perform a clustering robust standard error regression.

#### 3.3.2 Moderator effect model

To delve deeper into the working of the three variables of management shareholding, top management team stability, and executive power with an environmental protection background, we add three moderator variables to the baseline regression model interaction term for corporate green investment [56]. Following Hayes and Bolin, we centered the moderator variables to improve the explanatory power of the regression equation coefficients [90,91]. Moreover, considering the possible heteroscedasticity problem, this study regresses clustering robust standard on all three sets of moderating variables.

Equation (9) adds the moderator variable management shareholding (Mshare) and its product with the independent variable green investment to the base model of Equation (8) to measure the moderating effect of management shareholding. The model of Equation (9) refers to Javeed & Lefen's model [78] that uses management shareholding as a moderating variable as follows.

(9) $$Cei_{i,t}=\beta_{0}+\beta_{1}Cgi_{i,t}+\beta_{2}Mshare_{i,t}+\beta_{3}Cgi_{i,t}\times Mshare_{i,t}+\Sigma Control_{i,t}+\mu_{i}+\gamma_{t}+\varepsilon_{i,t}$$

Model (9) extends Model (8) by including the moderator variable, management shareholding (Mshare), and its interaction term with corporate green investment. For Model (9), if the coefficient on the interaction term is negative and statistically significant, management shareholding positively regulates the association between green investment and enterprise carbon emissions intensity.

Equation (10) refers to Meng et al.'s study of executive team stability as a moderating variable [92] by adding executive team stability and the cross-multiplier term between it and the independent variable to model (10).

(10) $$Cei_{i,t}=\gamma_{0}+\gamma_{1}Cgi_{i,t}+\gamma_{2}Stability_{i,t}+\gamma_{3}Cgi_{i,t}\times Stability_{i,t}+\Sigma Control_{i,t}+\mu_{i}+\gamma_{t}+\varepsilon_{i,t}$$

Model (10), based on Model (8), adds executive team stability (Stability) and its interaction term with corporate green investment. Assuming Model (10)'s interaction term coefficient is negative and statistically significant, the top management team's stability can further change the company's green investment carbon emission intensity reduction.

Equation (11) references Javeed & Lefen's study of CEO power as a moderating variable [78], and environmental context executive power is added as a moderating variable in model (11). At the same time, the cross-multiplier term between the moderating variable and the independent variable is added.

(11) $$\text{Ce}i_{i,t}=\theta_{0}+\theta_{1}\text{Cg}i_{i,t}+\theta_{2}\text{Epowe}r_{i,t}+\theta_{3}\text{Cg}i_{i,t}\times \text{Epowe}r_{i,t}+\Sigma \text{Contro}l_{i,t}+\mu_{i}+\gamma_{t}+\varepsilon_{i,t}$$

The moderating variable executive environmental background power (Epower) and its interaction item with the independent variable green investment of enterprises are added to Model (11). This model predicts that If there is a statistically significant negative coefficient for the interaction term, then the environmental protection background power of executives’ EPBP will strengthen enterprises’ positive role in increasing green investment to reduce their carbon emission intensity.

## 4 The outcomes of an empirical study

### 4.1 Statistical Descriptions and correlation analysis

Table 2 provides Statistical Descriptions for various variables.

**Table 2:** Statistical descriptions.

Variable	N	Mean	SD	Min	p50	Max
Cei	8034	0.0457	0.0158	0.0158	0.0435	0.1098
Cgi	8034	0.0452	0.0051	0.0337	0.0453	0.0572
Mshare	8034	0.2099	0.0361	0.1330	0.2105	0.2840
Stability	8034	0.5298	0.2004	0.1552	0.5127	0.9840
Epower	8034	0.5309	0.1992	0.1552	0.5170	0.9808
Size	8034	21.8013	1.0128	19.9660	21.6844	25.0386
Lev	8034	0.3747	0.1916	0.0485	0.3605	0.8438
Roa	8034	0.0457	0.0594	−0.1704	0.0433	0.2139
Tagr	8034	0.1574	0.2450	−0.2563	0.1000	1.1551
Cflow	8034	0.0465	0.0670	−0.1472	0.0450	0.2307
Tat	8034	0.6053	0.3414	0.0922	0.5379	2.0614
Top10	8034	0.3888	0.1677	0.1297	0.3600	0.8674
Soe	8034	0.0596	0.2368	0.0000	0.0000	1.0000
Age	8034	2.7765	0.3834	1.6094	2.8332	3.4657

For carbon intensity (Cei), the mean value stands at 0.0457 with a standard deviation of 0.0158, suggesting a normal distribution pattern for this variable. Its median value, at 0.0435, is slightly below the mean. This suggests that just under half of the enterprises have carbon emission intensities above the industry average, and the distribution of data is relatively symmetrical. The range for carbon emission intensity spans from a low of 0.0158 to a high of 0.1098, highlighting the variability of carbon emissions across different enterprises. Some enterprises exhibit particularly high carbon emissions.

The mean value for green investment (Cgi) in Chinese enterprises is 0.0452, with a standard deviation of 0.0051. This implies a generally positive trend in green investment across the sampled enterprises. The median value is closely matched to the mean at 0.0453, meaning that roughly 50 % of China's businesses have engaged in green investments. The data spread for this indicator ranges from 0.0337 to 0.0572, indicating variability in green investment levels across enterprises.

Management shareholding (Mshare) averages at 0.2099, with a standard deviation of 0.0361. The range for this variable is 0.1330–0.2840, with a median value of 0.2105. This indicates that more than half of the sampled firms have a management shareholding rate exceeding the industry average, though the highest shareholding rate does not surpass 30 %.

Regarding the stability of the executive team (Stability), the mean is at 0.5298, slightly higher than its median of 0.5127. Less than half of the sampled firms achieve stability levels at or above the industry average. The stability index ranges broadly, from 0.1552 to 0.9840, suggesting significant variations in executive team stability across firms.

The distribution characteristics of environmental background executive power (Epower) are akin to those of the executive team stability, showcasing a primarily positive trend.

In summary, the sample firms display considerable variability in their features. Notably, the data does not present significant outliers or values that would challenge the regression hypothesis. This indicates that the selected sample aligns well with the study's objectives.

To rule out multicollinearity, we calculate the Pearson correlation coefficients. Table 3 shows that corporate green investment (Cgi) and corporate carbon emission intensity (Cei) have a significant link with a coefficient of −0.319, indicating that the previous assumption is valid. All the correlation coefficients in Table 3 are less than 0.8, suggesting a high degree of independence between the variables. In addition, the vif value test shows that none of the factors have a vif value of more than three, ruling out the possibility of multicollinearity affecting the primary findings.

**Table 3:** Correlation matrix.

Variables	Cei	Cgi	Mshare	Stability	Epower	Size	Lev	Roa	Tagr	Cflow	Tat	Top10	Soe	Age
Cei	1													
Cgi	−0.319***	1												
Mshare	−0.256***	0.282***	1											
Stability	−0.276***	0.298***	0.258***	1										
Epower	−0.279***	0.283***	0.259***	0.749***	1									
Size	0.047***	−0.0170	−0.089***	0.079***	0.078***	1								
Lev	0.081***	−0.0120	−0.124***	−0.00400	−0.00800	0.498***	1							
Roa	0.029***	−0.027**	0.054***	−0.070***	−0.066***	−0.00300	−0.345***	1						
Tagr	0.170***	−0.065***	−0.00200	−0.120***	−0.113***	0.140***	0.090***	0.317***	1					
Cflow	−0.086***	0.021*	0.024**	0.098***	0.097***	0.030***	−0.186***	0.408***	−0.069***	1				
Tat	−0.114***	0.024**	−0.00900	−0.038***	−0.037***	0.046***	0.118***	0.272***	0.084***	0.202***	1			
Top 10	−0.00900	0.033***	−0.126***	−0.067***	−0.077***	0.046***	0.064***	0.0160	−0.078***	0.039***	0.056***	1		
Soe	0.063***	0.00700	−0.072***	−0.042***	−0.037***	0.106***	0.167***	−0.103***	−0.050***	−0.059***	−0.030***	0.059***	1	
Age	0.046***	0.00200	−0.046***	0.238***	0.247***	0.185***	0.157***	−0.087***	−0.078***	0.063***	−0.0160	0.00300	0.132***	1

Note: ***, **, and * indicate significance at 0.01, 0.05, and 0.1 levels of significance, respectively.

### 4.2 Analysis of empirical results

#### 4.2.1 The impact of corporate green investment on corporate carbon emission intensity

First, we conduct the Hausman test, and the p-value stands at 0.000. Then, we choose the fixed effects regression model. Corporate green investment and carbon emission intensity are regressed in Table 4. Column (8) of Table 4 reveals that, after accounting for a number of confounding variables, the regression coefficients of company green investment (Cgi) and corporate carbon emission intensity (Cei) is −0.7574, which is negative significant. This indicates a strong negative association, suggesting that companies reduce their carbon emission intensity as they invest more in green technologies. Thus, H1 receives support.

**Table 4:** Results of the regression analysis.

	(8)	(9)	(10)	(11)
Variables	Cei	Cei	Cei	Cei
Cgi	−0.7574***	−0.5567***	−0.5975***	−0.5845***
	(-16.9720)	(-12.7969)	(-13.5262)	(-12.9170)
Mshare		−0.1117***		
		(-13.7552)		
Mshare*Cgi		−0.0005***		
		(-2.6262)		
Stability			−0.0155***	
			(-10.8614)	
Stability*Cgi			−0.0019***	
			(-9.9515)	
Epower				−0.0179***
				(-11.7271)
Epower*Cgi				−0.0016***
				(-8.3436)
Size	−0.0022***	−0.0019***	−0.0019**	−0.0019***
	(-2.9939)	(-2.6643)	(-2.5138)	(-2.6245)
Lev	0.0051**	0.0048*	0.0053**	0.0048*
	(1.9735)	(1.8917)	(2.0624)	(1.8536)
Roa	0.0205 ***	0.0200 ***	0.0202 ***	0.0191 ***
	(4.0693)	(4.0609)	(4.1590)	(3.9318)
Tagr	0.0091 ***	0.0084 ***	0.0082 ***	0.0083 ***
	(9.5391)	(8.8085)	(8.7113)	(8.9864)
Cflow	0.0016	0.0017	0.0008	0.0010
	(0.3880)	(0.4224)	(0.2020)	(0.2508)
Tat	−0.0043 ***	−0.0046 ***	−0.0041 ***	−0.0037 ***
	(-3.3054)	(-3.4441)	(-3.3373)	(-3.0198)
Top 10	−0.0027	−0.0041 *	−0.0029	−0.0032
	(-1.1393)	(-1.6717)	(-1.2171)	(-1.3567)
Soe	0.0015	0.0010	0.0011	0.0010
	(0.9307)	(0.6260)	(0.7203)	(0.6611)
Age	0.0045	0.0030	0.0042	0.0042
	(1.5783)	(1.0576)	(1.5653)	(1.5419)
_cons	0.1162***	0.1299***	0.1066***	0.1079***
	(7.3507)	(8.3127)	(6.7545)	(6.9377)
Industry	Yes	Yes	Yes	Yes
Year	Yes	Yes	Yes	Yes
N	8034	8034	8034	8034
R^2^	0.115	0.158	0.179	0.181
adj. R^2^	0.113	0.155	0.176	0.179
F	29.3115	51.2690	77.8971	66.4256

Note: t statistics are in parentheses; *p < 0.1, **p < 0.05, ***p < 0.01.

#### 4.2.2 Moderating effect of management shareholding

After controlling a number of variables, corporate green investment (Cgi) in Model (9) is significantly negative (at the 1 % level) at −0.5567, and its interaction term with management shareholdings (Cgi*Mshare) is significantly negative (at the 1 % level) at −0.0005. According to the results, we knew the more money put into environmental initiatives by a company, the less damaging its carbon footprint will be. Moreover, this beneficial effect is enhanced even further by a rise in management's share, and the adj. R^2^ in Model (9) also elevated to a high level. Thus, H2 is supported.

#### 4.2.3 Moderating effect of top management team stability

As known in Column (10) of Table 4, the coefficient of the Cgi in Model (10) is −0.5975. The coefficient of (Cgi*Stability) is −0.0019. Further, both findings hold statistical significance. This demonstrates that the stability of the top management team can significantly strengthen the negative association between a company’s green investment and corporate carbon emission intensity. Furthermore, the adj. R^2^ in Model (10) increases dramatically to 0.176. Thus, H3 is supported.

#### 4.2.4 Moderating effect of environmental protection background executive power

From the regression results in Column (11) of Table 4, we see that the independent variable (Cgi) coefficient of Model (11) is −0.5845 and the coefficient of the intersection item (Cgi* Epower) is −0.0016, both of which are crucial. This shows that the greater the power of executives with environmental protection backgrounds, corporate green investment's impact on cutting carbon intensity increases as the investment is more substantial. The adj. R^2^ in Model (11) increased to 0.179. Therefore, H4 is supported.

### 4.3 Robustness check

#### 4.3.1 Removing 2020 samples for robustness testing amid the epidemic

As the COVID-19 swept the world in 2020, governments took steps to stem the virus's spread, such as shutting down businesses and restricting the movement of people, which led to a decline in economic growth [93]. The pandemic's impact also drove a global consensus on sustainable development, positioning funding for environmental sustainability as a pivotal strategy to promote the transformation of the global green economy [94]. This shift saw many countries intensifying their focus on green R&D and investments in renewable energy [95]. Given the potential disparities among enterprises in 2020 concerning production, operations, and green investment, this year could introduce deviations in research results, we choose to omit the sample data from 2020.

The coefficient of Cgi is −0.6373 after removing the sample data for 2020, which is statistically significant (Column 2 of Table 5). As expected from the regression results presented in Table 4, increasing green investments in enterprises can significantly reduce carbon emissions.

**Table 5:** Robustness test after removing 2020 samples.

	(1)	(2)
Variables	Cei	Cei
Cgi	−0.6597 ***	−0.6373 ***
	(-13.8428)	(-13.7837)
Cgi2		
Size		−0.0019 **
		(-2.3186)
Lev		0.0059 **
		(2.0652)
Roa		0.0266 ***
		(4.4566)
Tagr		0.0087 ***
		(8.5792)
Cflow		0.0020
		(0.4665)
Tat		−0.0044 ***
		(-3.1495)
Top 10		−0.0018
		(-0.6937)
Soe		0.0046**
		(2.2779)
Age		0.0053*
		(1.6565)
_cons	0.0778***	0.1020***
	(34.8105)	(5.7058)
Industry	Yes	Yes
Year	Yes	Yes
N	7003	7003
R^2^	0.065	0.096
adj. R^2^	0.064	0.093
F	31.0133	21.9572

Note: t statistics are in parentheses; *p < 0.1, **p < 0.05, ***p < 0.01.

#### 4.3.2 Robustness test after replacing the independent variable

Total green environmental protection capital and operating expenditures (Cgi) are the source of the study's green investment statistics, while academic circles often only use capital expenditures related to environmental protection [56]. Thus, we only use the green-related capital expenditure (Cgi2) as the independent variable for testing.

Column (2) of Table 6 shows the findings after controlling for a lot of factors and displays the substantial negative regression coefficient of −0.9501, meaning company green investment significantly reduces a company’s carbon emissions intensity, consistent with earlier regression studies.

**Table 6:** Robustness test.

	(1)	(2)
Variables	Cei	Cei
Cgi		
Cgi2	−0.9742 ***	−0.9501 ***
	(-18.1781)	(-18.3202)
Size		−0.0022 ***
		(-2.9064)
Lev		0.0052 **
		(1.9703)
Roa		0.0211 ***
		(4.1679)
Tagr		0.0091 ***
		(9.3940)
Cflow		0.0012
		(0.3094)
Tat		−0.0046 ***
		(-3.5174)
Top 10		−0.0030
		(-1.2574)
Soe		0.0016
		(0.9987)
Age		0.0041
		(1.4383)
_cons	0.0772***	0.1107***
	(43.1041)	(6.9582)
Industry	Yes	Yes
Year	Yes	Yes
N	8034	8034
R^2^	0.088	0.117
adj. R^2^	0.087	0.115
F	47.3672	32.3440

Note: t statistics are in parentheses; *p < 0.1, **p < 0.05, ***p < 0.01.

#### 4.3.3 Endogeneity test based on two-stage least squares method

Baseline regressions may have endogeneity issues owing to the study's omitted variables, measurement bias and reverse causation. Thus, we adopt TSLS for the endogeneity test, as described by Chen et al. [56]. For corporate green investment, we opt for lagged one-period data (lCgi), an independent variable proposed by Li et al. [96].

Table 7 displays the TSLS analysis outcomes. First-stage results show a statistically significant relationship between green investment and its instrumental counterpart. Second, at the 5 % level of significance, corporate green investment (Cgi) has a regression coefficient of −0.7231. The fact that the instrumental factors and regression results are in agreement with previous studies adds extra credence to the validity of this work. It is possible to identify the instrumental variable because the Kleibergen-Paap rk LM value is 58.15 (Table 7), and the corresponding p-value is 0. When applied to the Stock-Yogo weak ID test, the F value of Cragg-Donald Wald is much higher than the significance level of 16.38 needed to reject the null hypothesis. This disproves the hypothesis that there is an issue with inadequate instrumental variables.

**Table 7:** Two-stage least squares regression.

	(1)	(2)
Variables	Cgi	Cei
L.Cgi	−0.1099***	
	(-7.8865)	
Cgi		−0.7231**
		(-2.0326)
Size	−0.0003	−0.0029***
	(-1.1001)	(-3.2005)
Lev	−0.0005	0.0073**
	(-0.5945)	(2.3625)
Roa	−0.0035*	0.0246***
	(-1.9045)	(4.3609)
Tagr	−0.0008**	0.0084***
	(-2.1963)	(6.7944)
Cflow	0.0028*	0.0015
	(1.8014)	(0.3239)
Tat	−0.0001	−0.0041**
	(-0.1057)	(-2.5666)
Top10	0.0001	−0.0037
	(0.0711)	(-1.3399)
Soe	−0.0004	0.0018
	(-0.7480)	(1.0380)
Age	0.0008	0.0058*
	(0.6350)	(1.7410)
Industry	Yes	Yes
Year	Yes	Yes
Observations	6266	6185
R-squared	0.0211	0.1290
Number of id	1154	1073
r2_a	0.0181	−0.0577
F	5.6247	9.1947
Kleibergen–Paap rk LM statistic	58.15 (Chi-sq(1) p-val = 0.0000)	
Cragg–Donald F statistic	63.18	
Kleibergen – Paap rk Wald F statistic	62.20	
10 % maximal instrumental variable size	16.38	

Note: Robust t-statistics in parentheses ***p < 0.01, **p < 0.05, *p < 0.1.

## 5 Research results

Using the fixed effects model, this study concludes that for every additional unit of green investment in firms, the carbon emissions intensity of enterprises reduce by 0.0007574 units. The findings also stand the endogeneity and robustness tests. Thus, the first hypothesis is proven. Simultaneously, from the management standpoint, this study investigates the mechanism of green investment in firms’ carbon emission intensity. Furthermore, a causality analysis confirms that management shareholding, etc can further improve the impact of corporate green investment in reducing corporate carbon emission intensity. Thus, Hypotheses 2–4 are proven.

The results of the regression analysis are reported in Table 4 in this study and t-values (values in parentheses) are reported. Column (8) of Table 4 demonstrates the regression results of the base model. The regression coefficient of green investment (Cgi) on carbon intensity (Cei) is −0.7574, corresponding to a t-value of −16.9720, which is greater in absolute value than the Z-value at the 99 % confidence level, and is significant with three stars. Column (9) shows the results after adding the moderating variable management shareholding (Mshare) and the cross-multiplication term of Mshare with the independent variable green investment (Cgi). The coefficient of the cross-multiplication term, −0.0005, corresponds to a t-value of −2.6262, which is greater in absolute value than the Z-value at the 99 % confidence level and is significant with three stars. Column (10) shows the results after adding the moderator variable management shareholding (Stability) and the cross-multiplier term of Stability with the independent variable green investment (Cgi). The coefficient of the cross-multiplier term, −0.0019, corresponds to a t-value of −9.9515, which is greater in absolute value than the Z-value at the 99 % confidence level and is significant with three stars. Column (11) shows the results after adding the moderating variable management shareholding (Epower) and the cross-multiplier term of Epower with the independent variable green investment (Cgi). The coefficient of the cross-multiplier term −0.0016 corresponds to a t-value of −8.3436, which is greater in absolute value than the Z-value at the 99 % confidence level and is significant with three stars. The above results indicate that all the hypotheses are valid.

## 6 Discussion and conclusion

### 6.1 Discussion

We focus on the controversial issue of carbon emissions and explores the important factors affecting enterprises’ carbon emission intensity and their mechanisms of action. To alleviate environmental problems and realize low-carbon transformation, scholars from various countries are actively exploring the important factors affecting carbon emission reduction. For example, Ren [97] proved that an improvement in the green financial development index and an expansion of renewable energy sources will lessen the use of carbon. Raghutla et al. [98] looked for factors affecting carbon emissions from the standpoint of capital investment and found that foreign direct investment and R&D expenditures play a significant role in encouraging renewable energy usage. Multiple studies have shown that variables related to green topics have been the main focus of the exploration of the carbon emissions determinants.

Prior studies has shown that carbon reduction is significantly impacted by investment in renewable energy and sustainable development at the national level. Considering that enterprises are the main body of economic activity, it is more practical to study carbon emission factors in the corporate setting. Further, the achievement of business sustainable development objectives is heavily reliant on the course taken by corporate investment decisions [99]. According to previous research, non-fossil energy contributes significantly to cutting down on greenhouse gas production; thus, we chose to investigate the value of eco-friendly investments at the firm level in reducing carbon emissions. Chen and Ma [56] found that green investment can improve environmental performance of Chinese companies. Musibau [100], using quantile regression, demonstrated that increased investments in renewable energy can reduce greenhouse gas emissions and boost environmental quality. Micro-level studies collected at the provincial level have shown that green investment is beneficial to the environment. Therefor, this study considers corporate green investment as a research variable and uses corporate-level data to explore its impact on corporate carbon emission. The empirical analysis results also prove that enterprises can drastically lower the intensity of their carbon emissions by increasing green investment and compensating for this gap at the micro level.

In addition, scholars have explored its role in greenhouse gas emissions from the perspective of corporate governance, because companies can lower their carbon footprints and climate risks through the implementation of appropriate governance procedures [101]. Many academics have recognized management as a vital facet of environmental management. For example, Luo et al. proved that management awareness of carbon risk significantly influence corporate governance's impact on carbon performance [102]. Management shareholding, as an incentive, is considered to help companies participate in more corporate social responsibility activities [103], such as green innovation and active carbon emission reduction. Regarding the economic consequences of top management team stability, relevant studies have found that it affects a company's financial performance, technological innovation efficiency, and innovation investment level from the internal perspective of the enterprise [104,105]. Some scholars have proven that, under the adjustment of executive power, company social responsibility and company performance have a considerable positive link [106]. Previous studies have highlighted the importance of management's role in corporate green and low-carbon behaviors. However, current research still lacks a comprehensive understanding of the mechanisms impacting corporate green investment behavior and carbon emission reduction behavior from the perspectives of management shareholding, senior management team stability, and executive influence with an environmental background. Further, this study examines the managerial perspective on what the mechanism is.

With the global emphasis on ecology, scholars have begun to study topics related to carbon emissions. Scholars have begun to explore the relationship between green investment and carbon emissions at the national level. Sharif et al. used data from G7 countries over a period of 24 years to make an argument and found that green finance has a negative and significant impact on carbon dioxide [107]. Ren et al. also studied the relationship between green investment and carbon emissions using data from 30 provinces in China and found that Green investment can reduce environmental pollution by improving energy-saving and emission reduction efficiency and enhancing innovation capacity [25]. Although many scholars choose the macro perspective to explore the relationship between green investment and carbon emissions, the findings of negative correlation between the two also provide inspiration for this study. Because enterprises are the main players in environmental pollution and protection, it is also crucial to explore the relationship between the two at the enterprise level. Using publicly disclosed information such as corporate annual reports and sustainability reports, this study manually collected data on corporate green investment and carbon emissions and analyzed them using a high-dimensional fixed effects model. The results of the analysis concluded that enterprises can reduce their carbon emission intensity through green investment.

Although previous studies on carbon emissions have mostly focused on the national level, some scholars have also started to analyze them at the micro level. Liu et al. have found that there is a positive correlation between the profitability pressure of Chinese enterprises and the intensity of sulphur dioxide emissions, which to some extent reveals the conflict between enterprises' financial goals and environmental goals. However, as a major participant in social production activities, enterprises should not only care about short-term financial goals, but also focus on their own sustainable development. Investment decisions, as a means to achieve long-term corporate goals, are also worth using to study the relationship between them and corporate environmental goals or even sustainable development goals. Zhan & Santos-Paulino studied that public sector investment is still the main source of funding to achieve the sustainable development goals, and private investment is crucial to achieve the goal [108]. As countries around the world are pursuing green development, the concept of green investment has emerged. For China, green investment has become a major driver for its green development [25]. Does green investment at the enterprise level have the same driving force for its green transformation of energy saving and emission reduction? Many scholars have conducted research on R&D investment, innovation investment, etc. Safitri et al. argued that R&D investment is conducive to improving the eco-efficiency of enterprises [109], while Albitar et al. explored the relationship between corporate environmental innovation and carbon emissions, and came to the conclusion that corporate environmental innovation can reduce corporate carbon emissions [87]. This study is not limited to the enterprise's investment in technological innovation but is based on the capitalized and expensed expenditures related to the enterprise's environmental protection to explore the relationship between green investment and the enterprise's carbon emissions, and the conclusions of the study will be a clearer guidance for the enterprise's investment decision. In addition, according to previous studies, the agglomeration effect of corporate carbon emission intensity is reflected in regional carbon emission intensity. Yu et al. argued that the diseconomies of scale problem caused by excessive economic agglomeration leads to the increase of regional carbon intensity [110]. Liu et al. argued that there is an inverted U relationship between industrial agglomeration and carbon emissions, and that carbon emission reduction is realized through technological innovation [111]. This study will get rid of the regional problem of carbon emission, collect carbon emission data from the enterprise level, and explore the relationship between enterprise green investment and carbon emission intensity under the premise of fully considering the individual effect and industry effect, in order to get more universal research results at the enterprise level.

In addition, previous studies have often studied and understudied management-related metrics, such as diversity in executive tenure and board gender, as determinants of corporate carbon emissions. Research results show that tenure diversity leads to less effective reduction of corporate carbon emissions [112], and board gender diversity is more responsive to stakeholder needs and reduces corporate carbon footprint [113]. In addition, regarding the management side, there are some prior studies that have explored its related variables as important moderating variables. For example, Ang et al. have argued that CSR can positively affect the corporate financial performance of heavily polluting listed companies in China. However, ownership concentration reduces this positive impact [114]. Javeed & Lefen similarly discussed the relationship between CSR and firm performance and added CEO power and ownership structure as moderating variables. By collecting and analyzing data from Pakistani firms, the authors concluded that both moderating variables positively moderated the positive relationship between CSR and firm performance [78]. There is a lack of clarity regarding the relationship of management ownership on corporate environmental responsibility and financial performance, but what is certain is that it plays an important role in achieving the financial and environmental objectives of the firm. Instead of focusing on the performance issue, which has been studied by many scholars, this study takes energy saving and emission reduction as the environmental goal and continues to explore what role the issue of management ownership plays in the realization of environmental goals by enterprises. This will also provide a strong basis for subsequent investment decisions and the achievement of environmental goals. Meng et al. explored the relationship between "ownerless" firms and environmental performance in the absence of a beneficial owner. The study concluded that there is a positive relationship between the two due to legitimacy considerations, and by adding executive team stability as a moderating variable, the study showed that TMT has a negative moderating effect on the two [92]. This study not only considers the management team as an important object of financial decision-making, but also as an important object of environmental decision-making. The results better reflect the important role played by the management team in the green production, operation and decision-making of the whole chain of enterprises, and provide an important direction for enterprises to realize the goal of green and sustainable development. In this study, from the perspective of moderating variables, management shareholding, executive team stability and environmental background executive power are included in the analytical framework of the theme of green investment and carbon emission, and the empirical analysis obtains fresh conclusions. The study finds that all three of these enhance the role of firms in reducing carbon emissions through green investment. This also provides a feasible path for companies to further focus on management and utilize managers to achieve efficient energy saving, emission reduction, and green transformation goals.

#### 6.1.1 Research significance

This study delves into both conceptual and pragmatic implications surrounding factors that influence corporate carbon emission reduction, promoting a push for corporations to increase green investment and actively pursue green transformation.

On the theoretical front, this study broadens the scope of studies examining carbon emissions from corporations and provides new contexts in which to apply themes from environmental governance. It also holds significant theoretical relevance from a micro-enterprise standpoint. Establishing a link between corporate green investment and carbon emissions reduction creates a path for businesses to proactively engage in green transformation and investment. This, in turn, promotes the realization of carbon emissions reduction and fosters a green, low-carbon economy. From a practical standpoint, empirical evidence underscores that corporate green investment significantly influence businesses to curtail their carbon footprints, offering an efficient route for businesses to transition to a low-carbon model. Elevating green investment not only encourages businesses to earnestly fulfill their environmental and social responsibilities but also helps them overcome management shortsightedness and attain sustainable growth. Recognizing that executives are instrumental in a company's decision-making, this study's findings offer several policy recommendations:

Promotion of equity incentives is vital, focusing on refining the methods of incentive. Companies should progressively establish and enhance equity incentive mechanisms tied to environmental and social responsibility and sustainable development outcomes. The transition of equity incentive pathways from theoretical to practical is also essential. Concurrently, regulations should allow for more flexibility in management shareholding, thereby optimizing the efficiency of equity incentive policies.

Ensuring stability within the top management team is crucial. This team represents a company's most valuable human capital. A consistent senior management team not only curtails operating costs but profoundly influence a company's commitment to social responsibilities and sustainable growth. As such, firms should exercise prudence in management appointments and terminations. They should also craft salary contracts that balance incentives and constraints to attract and retain elite executives. Full transparency about company personnel changes, including reasons, impacts, and countermeasures, is necessary to mitigate information asymmetry with external stakeholders, preventing unwarranted concerns that might disrupt the company's regular operations.

A dynamic management structure that aligns with the company's strategic objectives and environmental considerations is essential, necessitating optimal human resource management. To align with domestic and global low-carbon transformation trends, the power dynamics within the senior management team should exhibit flexibility. Executing a green transformation strategy demands leaders who prioritize environmental issues. Beyond recruiting and nurturing such executives, mechanisms that empower them to leverage their expertise fully are indispensable. This ensures harmony between talent, strategies, and organizational systems.

Lastly, governmental bodies can implement green financial policies to alleviate financial strains on companies, ensuring they have the fiscal backing to augment their green investments. By broadening its support for businesses' green and low-carbon endeavors, the government can stimulate their enthusiasm for green innovation investment, cleaner production, and energy conservation. This can be achieved by offering financial subsidies, tax incentives, and other perks to businesses contributing to green innovation, cleaner production, and emission reduction initiatives.

### 6.2 Conclusion

#### 6.2.1 Research conclusions

As the global climate problem is becoming more and more serious, carbon emission reduction, energy transition and green sustainable development have become a hot topic of discussion [115]. However, the issue of factors influencing carbon emissions has not yet been conclusively determined and how China solves the carbon emission problem has received global attention. In view of this, this study is dedicated to exploring the determinants of carbon emissions of Chinese enterprises, and selects the carbon intensity of Chinese enterprises as a measure of the extent of corporate carbon emissions. At the same time, corporate green investment is chosen as the main object to explore the factors influencing carbon emissions. Because of the corporate perspective, and management plays an inaccessible position in corporate business decisions, this study intends to explore whether green investment can have an impact on corporate carbon emission intensity while further exploring what kind of role management plays in the process. After controlling for a series of influential factors that may affect carbon emission intensity, this paper utilizes a high-dimensional fixed effect model to empirically analyze the research question. The results of the regression analysis show that green investment is significantly negatively correlated with corporate carbon intensity at the 1 % significance level. According to the regression coefficient, it can be seen that for every unit increase in green investment, the carbon emission intensity of enterprises is reduced by 0.0007574 units. In addition, this study added the cross-multiplier terms of the three moderating variables and the independent variable green investment respectively in the base model, and the coefficients of the cross-multiplier terms were all significantly negative at the 1 % significance level. It indicates that management shareholding, executive team stability and environmental background executive power can all increase the negative impact of green investment on corporate carbon intensity. All hypotheses in this thesis are verified to be valid. Moreover, the findings of this study are still consistent with the previous study by changing the sample time, replacing the independent variables and using the 2SLS method. This further argues the importance of green investment in realizing corporate carbon emission reduction. The findings of the study provide direction and guidance for enterprises' investment decisions, and moreover, it triggers enterprises to pay more attention to the management, and it also strengthens enterprises' goal of energy saving and emission reduction.

#### 6.2.2 Limitations and future directions

At present, we exclusively examines the impact of corporate green investment on carbon emissions reduction. Nonetheless, numerous internal and external factors, such as government public financial support, levels of green financial development, corporate blockchain technology, and advancements in artificial intelligence, potentially influence corporate carbon emissions reduction. Future empirical analyses can delve into the specific relationships between these factors and corporate carbon emissions reduction, thereby enriching the discourse on corporate carbon emissions.

In measuring corporate greenhouse gas emissions, this study considers only the carbon emissions specified in the Greenhouse Gas Protocol for its statistical scope. Emissions connected to activities along the value chain are not incorporated. In contrast, the European Union has proffered non-binding guidelines for climate-related data reporting, heavily endorsing the disclosure of it. Going forward, econometric techniques can produce comprehensive statistics on the full scope of corporate carbon emissions. This will solidify the pivotal role of corporate green investment in carbon emissions reduction across the entire industrial chain. Concurrently, the metrics for calculating corporate carbon emissions can be broadened beyond merely carbon emission intensity, exploring other facets such as carbon management proficiency and low-carbon revenue.

This study adopts a managerial viewpoint to probe the direct link between corporate green expenditure and a company's carbon emissions intensity. Executive compensation in the study hinges on tournament theory, which possesses an inherent incentive impact on executives. Future research can widen the lens on management, encompassing areas like environmental consciousness among executives, executive salary disparities, and instances of excessive executive compensation. Moreover, through the lens of corporate governance and external oversight, there's potential to delve deeper into variations in corporate green investment and its subsequent effects on carbon emissions intensity, leading to more nuanced findings.

## Funding statement

This paper has no funding affiliation to declare.

## Data availability statement

Data will be made available on request.

## CRediT authorship contribution statement

**Sisi Zheng:** Writing – original draft, Investigation, Data curation, Conceptualization. **Shanyue Jin:** Writing – review & editing, Methodology, Investigation, Conceptualization.

## Declaration of competing interest

The authors declare the following financial interests/personal relationships which may be considered as potential competing interests:
